# The prognostic impact of lymphocyte-to-C-reactive protein score in patients undergoing surgical resection for intrahepatic cholangiocarcinoma: A comparative study of major representative inflammatory / immunonutritional markers

**DOI:** 10.1371/journal.pone.0245946

**Published:** 2021-01-28

**Authors:** Daisuke Noguchi, Naohisa Kuriyama, Yuki Nakagawa, Koki Maeda, Toru Shinkai, Kazuyuki Gyoten, Aoi Hayasaki, Takehiro Fujii, Yusuke Iizawa, Akihiro Tanemura, Yasuhiro Murata, Masashi Kishiwada, Hiroyuki Sakurai, Shugo Mizuno

**Affiliations:** Department of Hepatobiliary Pancreatic and Transplant Surgery, Mie University Graduate School of Medicine, Tsu, Mie, Japan; Texas A&M University, UNITED STATES

## Abstract

**Background:**

In many malignancies including intrahepatic cholangiocarcinoma (iCCA), prognostic significance of host-related inflammatory / immunonutritional markers have attracted a lot of attention. However, it is unclear which is the strongest prognostic indicator for iCCA among these markers. The aim of this study was to firstly evaluate the prognostic utility of inflammatory / immunonutritional markers in resected iCCA patients using a multiple comparison in addition to a new marker, lymphocyte-to-C-reactive protein (CRP) score.

**Methods:**

A total of sixty iCCA patients, who underwent surgical resection between October 2004 and April 2019, were enrolled in this study. Their clinical and pathological data were retrospectively assessed using univariate and multivariate analysis to determine prognostic predictors for disease specific survival (DSS). Moreover, these patients, who were divided into high and low groups based on lymphocyte-to-CRP score, were compared these survival outcomes using Kaplan-Meier analysis with a log-rank test.

**Results:**

In multivariate analysis, the significant prognostic factors were preoperative lymphocyte-to-CRP score (p = 0.008), preoperative CRP-to-albumin ratio (CAR; p = 0.017), pathological T category (p = 0.003), and pathological vascular invasion (p < 0.001). Resected iCCA patients with a low lymphocyte-to-CRP score (score 0) had significant better prognosis than patients with a high score (score 1 or 2) (p = 0.016). Notably, the mortality of the high lymphocyte-to-CRP score group did not show statistically difference from the poor mortality of unresected iCCA patients (p = 0.204).

**Conclusions:**

Preoperative lymphocyte-to-CRP score was the strongest prognostic indicator in iCCA patients with surgical resection. In these patients, early intervention with nutritional support should be considered prior to operation.

## Introduction

Intrahepatic cholangiocarcinoma (iCCA) remains the second most frequent primary liver cancer following hepatocellular carcinoma (HCC) [1]. Surgical resection is considered the only choice of potentially curative treatment for patients with operable iCCA [2, 3]. Recently, as the number of iCCA patients is increased, demand for risk factors, early diagnostic markers and prognostic factors in iCCA have been increased.

In patients with malignancy including iCCA, the commonly acknowledged factors, which may serve as predictors for survival, are tumor size, lymph node metastasis, vascular invasion, and so on [4]. On the other hand, importance of nutritional deficiency and continuous systemic inflammation, which greatly contribute to tumor growth, invasion and metastasis, have been payed much attention [5, 6]. Over the past decade, several parameters and scoring systems have been developed based on some markers reflecting patient’s nutritional status and systemic inflammation, which are represented by the neutrophil to lymphocyte ratio (NLR) [7], the platelet to lymphocyte ratio (PLR) [8], the lymphocyte-to-monocyte ratio (LMR) [9], the prognostic nutritional index (PNI) based on albumin concentration and lymphocyte count [10], the C-reactive protein (CRP) to albumin ratio (CAR) [11], the Glasgow Prognostic Score (GPS) based on serum CRP and albumin [12], and the Controlling nutritional status (CONUT) score based on serum albumin, total cholesterol, and peripheral lymphocyte counts [13]. Moreover, accumulating studies demonstrated the potential of inflammatory / immunonutritional markers as good prognostic predictors in patients with malignancy. Recently. a new inflammatory / immunonutritional marker, lymphocyte-to-CRP score, was developed by Okugawa et al. [14] in 2020, according to an investigation for the best combination of systemic inflammatory factors derived from NLR, PLR and CAR [15]. Furthermore, he demonstrated that preoperative lymphocyte-to-CRP score was an independent prognostic factor for both overall survival and disease-free survival in gastric cancer patients. In addition to the well-established evidences of CRP as a systemic inflammatory marker [6], lymphocytes have also been used to assess immunonutritional status [16]; therefore, lymphocyte-to-CRP score is considered to reflect a status of not only inflammation but also immunonutrition in patients with malignancies.

As for iCCA patents, some previous studies elucidated an association between preoperative inflammatory / immunonutritional markers and survival outcomes. According to these studies for iCCA, high levels of NLR [17, 18], high levels of PLR [19], low levels of LMR [20], low levels of PNI [21], high levels of CAR [22], a high GPS score (score 1 / 2) [23], and a high CONUT score (more than score 2) [24] reflected poor overall survival after surgical resection. However, to our knowledge, there is no study to clarify which is the strongest prognostic indicator for resected iCCA patients among these markers. The aim of this study was to evaluate the prognostic utility of inflammatory / immunonutritional markers in iCCA patients with surgical resection, using a multiple comparison among all these markers, which had already been confirmed these prognostic significances for resected iCCA patients, in addition to a new marker, lymphocyte-to-CRP score.

## Materials and methods

### Patient selection

Between October 2004 and April 2019, sixty iCCA patients who underwent surgical resection in Mie university hospital were enrolled in this study. All selected patients were diagnosed as iCCA pathologically. They had sufficient clinical data available to evaluate inflammatory / immunonutritional markers before operation and complete long-term follow-up records. Their clinical and follow-up information was extracted from a database at Mie university hospital, and verified by reviewing patient medical records at Mie university hospital, and available for retrospective analysis. The day of the final follow-up was 30th June 2020. Additionally, fifty-eight unresected iCCA patients, who were not in a candidate for surgery because of multiple tumor location, extrahepatic metastasis, or huge tumor at our institution in the same period, were compared to resected patients. This study protocol was approved by the medical ethics committee of Mie University Hospital (No. H2018-073) and was performed in accordance with the tenets of the 1964 Declaration of Helsinki. The participants who gave consent to the present study by participating had the option of opt-out which was fully explained. All data were fully anonymized before we accessed under management by the ethics committee.

### Preoperative management

The 64-slice multidetector computed tomography (MDCT), magnetic resonance imaging (MRI), magnetic resonance cholangiopancreatography (MRCP), and endoscopic retrograde cholangiography (ERC) were used for preoperative diagnosis and tumor staging. Endoscopic retrograde biliary drainage (ERBD) tubes were inserted into the future remnant liver in patients with obstructive jaundice. In all enrolled patients, we confirmed that a center of tumor was located in the periphery of hilar bile duct, and evaluated presence or absence of tumor extent to hilar bile duct with preoperative image studies. Based on UICC 8^th^, hilar bile duct was defined as the duct located between the right side of the umbilical portion of the left portal vein and the left side of the origin of the right posterior portal vein [25–27].

Several patients with tumor extension to major vessels such as portal vein (PV), hepatic artery (HA), or regional lymph node metastasis were treated with preoperative chemotherapy. As for protocol of preoperative chemotherapy, gemcitabine-single regimen (1000 mg/body on days 1 and 15 every 4 weeks) were mainly used. From 2010, gemcitabine-based regimen plus S-1 (gemcitabine; 800 mg/m^2^ on days 8 and 22, S-1; 80 mg/body daily on days 1–21 every 6 weeks) or plus cisplatin (gemcitabine; 1000 mg/m^2^ on days 1 and 8, cisplatin; 25 mg/m^2^ on days 1 and 8 every 4 weeks) were used as a combination therapy. After re-evaluation, in the patients who could not receive curative-intent surgery, chemotherapy was continued.

### Surgical procedure

Based on the following factors: the indocyanine green retention rate at 15min (ICGR15), the hepatic uptake ratio of 99mTc- GSA scintigraphy at 15min (LHL15), and the future remnant liver volume using CT volumetry [28], the type of hepatectomy was determined according to Couinaud’s classification. Moreover, to obtain curative resection, a combined resection was added to hepatectomy depending on the biliary and vascular factors. In several patients with tumor extension to perihilar bile duct and/or regional lymph node metastasis, caudate lobectomy and extrahepatic bile duct resection as lymphadenectomy were additionally performed. In terms of a vascular invasion to major vessels in hepatic hilum, when at least 5 mm tumor free margin of PV and/or HA in the remnant liver side was secured, combined resection and reconstruction of invaded vessels was added. Partial hepatectomy was performed due to poor patient condition such as old age and insufficient remnant liver function. Meanwhile, when patients had insufficient future remnant liver volume according to preoperative CT volumetry, percutaneous transhepatic portal vein embolization (PTPE) was performed followed by hepatectomy. Surgical curability was defined by intraoperative and pathological tumor-free surgical margins.

### Follow-up survey and data collection

The follow-up surveys were conducted every 1–3 month within 3 years after surgery, half-yearly between 3 and 5 years, and every 1 year thereafter. The survey included comprehensive medical history, blood examination including tumor markers such as serum carcinoembryonic antigen (CEA) and carbohydrate antigen 19–9 (CA 19–9), and imaging surveillance by chest and abdominal computed tomography (CT). Survival was calculated in months from the date of surgery to death. The follow-up was continued for 120 months (mean 20.5 months).

### Biochemical parameters, inflammatory / immunonutritional markers and histology

**Fig 1:**
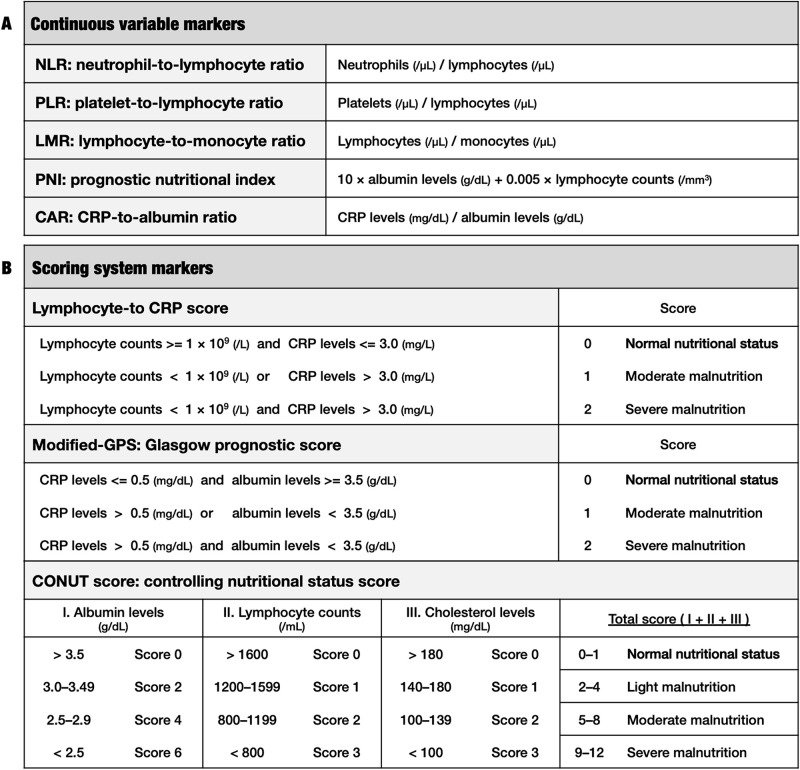
Definitions of inflammatory / immunonutritional markers. (A) Continuous variable markers. (B) Scoring system markers. Normal nutritional status of patients corresponds to low score as follows: score 0 in lymphocyte-to-CRP score and modified-GPS, and score 0 to 1 in CONUT score. Abbreviations: CRP: C-reactive protein.

Blood samples from each patient were obtained within 1 week prior to the surgical resection of their primary tumor for measurement of tumor markers (CEA and CA19-9), total bilirubin, total protein, albumin, cholesterol, C-reactive protein (CRP), white blood cell count (WBC), neutrophil count, lymphocyte count, monocyte count, and platelet count relevant to this study. Based on previous studies, inflammatory / immunonutritional markers, which were previously demonstrated these prognostic significances for resected iCCA patients, were obtained as follows: neutrophil-to-lymphocyte ratio (NLR) [17, 18, 29–32], platelet-to-lymphocyte ratio (PLR) [19, 31], lymphocyte-to-monocyte ratio (LMR) [20, 29, 31], prognostic nutritional index (PNI) [21, 33], CRP-to-albumin ratio (CAR) [22], modified-Glasgow prognostic score (GPS) [23], controlling nutritional status (CONUT) score [24]. Additionally, the lymphocyte-to-CRP score was also examined [14, 15]. Definitions of these markers were demonstrated at Fig 1. Pathological tumor staging and regional lymph node metastasis were defined according to Union for International Cancer Control (UICC) 8th edition.

### Data analysis

Continuous and categorical variables were expressed as median (interquartile range) and were compared using the Mann-Whitney U test and the Chi-square test. Patients who were alive or had died of a cause other than iCCA were censored for analysis of disease-specific survival (DSS). Survival was calculated using the Kaplan-Meier method and was compared between the groups using the log-rank test. The day of the final follow-up was 30th June 2020, at which time there was no loss of follow-up. Individual variables with a significance of p value < 0.05 in the univariate Cox proportional hazards model were selected for inclusion in the multivariate analysis. These factors affecting DSS were analyzed using the multivariate Cox proportional hazards model. A cut-off value for CAR was determined using a web-based software tool (Evaluate Cutpoints; http://wnbikp.umed.lodz.pl/Evaluate-Cutpoints/), and dichotomized for the analyses. All statistical analysis was performed using IBM SPSS Statistics for Macintosh (version 27; IBM Corp., Armonk, NY, USA). P value < 0.05 was considered statistically significant.

## Results

### Patient’s characteristics

Preoperative characteristics in sixty patients, who underwent surgical resection for iCCA, were shown in Table 1. The median age of patients was 67.5 years (range 60–75 years). Thirty-eight patients (63%) were male and twenty-two patients (37%) are female. Sixteen patients (26%) received preoperative chemotherapy. Six patients (10%) underwent PTPE and four patients (7%) received preoperative bile duct drainage prior to resection. Preoperative CT showed tumor extension to perihilar bile duct in thirty-four patients (57%). Preoperative inflammatory / immunonutritional markers were categorized into inflammation-based markers such as NLR, PLR, LMR, lymphocyte-to-CRP score and inflammation-nutrition-based markers such as PNI, CAR, modified-GPS, and CONUT score. Median values of NLR, PLR, LMR, PNI, and CAR were shown as follows: NLR was 2.2 (range 1.6–3.3), PLR was 125.7 (range 93.0–161.9), LMR 3.3 (range 2.6–4.9), PNI 47.6 (range 44.0–50.6), CAR 0.05 (range 0.02–0.16). In scoring systems of inflammatory / immunonutritional markers, low scores corresponded to normal nutritional status as shown in Fig 1. As for lymphocyte-to-CRP score, thirty-three (55%) patients were scored 0 (normal), twenty-one (35%) or six (10%) patients were scored 1 or 2. As for modified-GPS, thirty-nine (65%) patients were scored 0 (normal), and eighteen (30%) or three (5%) patients were scored 1 or 2. As for CONUT score, six (10%) or twenty (33%) patients were scored 0 or 1 (normal), twenty (33%), and five (8%), or six (10%) patients were scored 2, 3, or 4. There was one patient (2%) for each scored 5, 7, or 9.

**Table 1:** Preoperative characteristics in resected patients.

Variables	Patients (n = 60)
Age (y.o.)	67.5 [60–75]
Gender (Male / Female)	38 / 22
Preoperative therapy (yes / no)	
Chemotherapy	16 (26%)
PTPE	6 (10%)
Biliary duct drainage	4 (7%)
Preoperative finding of tumor extension to perihilar bile duct*	34 (57%)
Preoperative blood examination	
WBC (/μL)	5295 [4537–6357]
Neutrophil (/μL)	3090 [2507–4552]
Lymphocyte (/μL)	1415 [1140–1795]
Monocyte (/μL)	380 [287–515]
Platelet (x10^3^/μL)	18.3 [14.6–23.0]
Total bilirubin (mg/dL)	0.7 [0.5–1.0]
Total protein (g/dL)	7.1 [6.7–7.4]
Albumin (g/dL)	4.0 [3.7–4.2]
Cholesterol (mg/dL)	184.0 [148.7–206.5]
CRP (mg/dL)	0.20 [0.08–0.68]
CEA (ng/mL)	3.9 [2.0–6.4]
CA19-9 (U/mL)	70.1 [23.0–172.9]
Preoperative inflammation-based markers	
NLR	2.2 [1.6–3.3]
PLR	125.7 [93.0–161.9]
LMR	3.3 [2.6–4.9]
Lymphocyte-to-CRP score: 0 / 1 / 2	33 / 21 / 6
Preoperative inflammation-nutrition-based markers	
PNI	47.6 [44.0–50.6]
CAR	0.05 [0.02–0.16]
modified-GPS: 0 / 1 / 2	39 / 18 / 3
CONUT score: 0 / 1 / 2 / 3 / 4 / 5 / 6 / 7 / 8 / 9	6 / 20 / 20 / 5 / 6 / 1 / 0 / 1 / 0 / 1

* This finding was evaluated by preoperative computed tomography.

PTPE: percutaneous transhepatic portal vein embolization, WBC: white blood cell, CRP: C-reactive protein, CEA: carcinoembryonic antigen, CA19-9: carbohydrate antigen 19–9, NLR: Neutrophil-to-lymphocyte ratio, PLR: Platelet-to-lymphocyte ratio, LMR: Lymphocyte-to-monocyte ratio, PNI: Prognostic nutritional index, CAR: CRP-to-albumin ratio, GPS: Glasgow prognostic score, CONUT score: Controlling nutritional status score.

Inoperative and postoperative characteristics and histology in sixty resected patients were shown in Table 2. The most common type of liver resection was left hepatectomy (S2, 3, 4) in 11 patients (18%) with caudate lobectomy or thirteen patients (22%) without it. Regarding combined resection with hepatectomy, extra bile duct resection was performed in twenty-seven patients (45%), caudate lobectomy was performed in twenty-eight patients (47%), and PV or HA was combinedly resected followed by reconstruction in thirteen patients (22%). The median amount of blood loss was 1160 g (range 412–1950). The median operative time was 454 minutes (range 354–596). As for histology, thirteen patients (22%) had multiple tumors. The median value of maximal tumor diameter was 4.5 cm (range 3.1–6.6). Based on UICC 8th edition, Stage 0 to 2 patients, whose tumor is located within liver without lymph node metastasis, were two (3%) in Stage 0, seven (12%) in Stage1a, four (67%) in Stage 1b, and twenty-five (42%) in Stage 2. On the other hand, six patients (10%) were in Stage 3a, fourteen patients (23%) were in Stage 3b, and two patients (3%) were in Stage4. In terms of pathological T category (pT), pT3 to 4 patients, who had extrahepatic tumor extension or tumor invasion to adjacent organs, were ten (17%) in pT3 or one (2%) in pT4. Nine patients (15%) were in pT1a, four patients (67%) were in pT1b, and thirty-two (37%) patients were in pT2. Regional lymph node metastasis was shown in fifteen patients (25%), and distant metastasis was shown in two patients (3%). Eleven patients (18%) had visceral peritoneum invasion, fifteen patients (25%) had vascular invasion. Intrahepatic metastasis was shown in seven patients (12%). R0 resection was achieved in 48 patients (78%). Adjuvant chemotherapy was performed in twenty-eight patients (47%).

**Table 2:** Inoperative and postoperative characteristics and histology in resected patients.

Variables	Patients (n = 60)
Type of liver resection*	
S2, 3, 4, 5, 8 + S1	5
S4, 5, 6, 7, 8 + S1	2
S2, 3, 4 + S1	11
S2, 3, 4	13
S5, 6, 7, 8 +S1	9
S5, 6, 7, 8	4
S4, 5, 8 + S1	1
S4, 5, 8	1
S2, 3	4
S6, 7	1
Subsectionectomy	4
Partial hepatectomy	5
Combined resection	
Extra bile duct	27 (45%)
Caudate lobe	28 (47%)
Blood vessels (portal vein or hepatic artery)	13 (22%)
Blood loss (g)	1160 [412–1950]
Operative time (min)	454 [354–596]
Tumor number (single / multiple)	47 / 13
Maximal tumor diameter (cm)	4.5 [3.1–6.6]
TNM (UICC 8th)	
Stage: 0 / 1a / 1b / 2 / 3a / 3b / 4	2 / 7 / 5 / 24 / 6 / 14 / 2
pT: is / 1a / 1b / 2 / 3 / 4	2 / 10 / 5 / 32 / 10 / 1
pN: 0 / 1	45 / 15
M: 0 / 1	58 / 2
Visceral peritoneum invasion	11 (18%)
Vascular invasion	15 (25%)
Intrahepatic metastasis	7 (12%)
R0 resection	47 (78%)
Adjuvant chemotherapy	28 (47%)

*Expressed as Couinaud’s hepatic segments resected.

pT: pathological T, pN: pathological N.

### Univariate and multivariate analysis of factors contributing to DSS

As shown in Table 3, univariate analysis revealed the independent prognostic factors contributing to DSS in iCCA patients undergoing surgical resection as follows. WBC (p = 0.021), neutrophil (p = 0.002), CRP (p = 0.045), and CEA (p = 0.044) were extracted from preoperative blood examinations. NLR (p = 0.007), LMR (p = 0.022), lymphocyte-to-CRP score (score 0 vs 1 / 2, p = 0.022), PNI (p = 0.035), CAR (p = 0.048), and modified-GPS (0 vs 1 / 2, p = 0.009) were extracted from preoperative inflammatory / immunonutritional markers. Blood loss amount (p = 0.011), operative time (p = 0.021), pathological stage based on UICC 8th edition (0 / 1a / 1b / 2 vs 3a / 3b / 4, p = 0.040), pathological T category (is / 1a / 1b / 2 vs 3 / 4, p < 0.001), pathological visceral peritoneum invasion (p < 0.001), and pathological vascular invasion (p = 0.047) were extracted from postoperative characteristics. Multivariate analysis identified preoperative lymphocyte-to-CRP score (p = 0.008, HR 8.566, 95% CI 1.762–41.636), preoperative CAR (p = 0.017, HR 2.762, 95% CI 1.198–6.367), pT category (p = 0.003, HR 16.546, 95% CI 2.679–102.175) and pathological vascular invasion (p < 0.001, HR 18.459, 95% CI 3.990–85.391) as independent prognostic factors for DSS. Prognostic factors which could be identified preoperatively were only inflammatory / immunonutritional markers such as preoperative lymphocyte-to-CRP score and CAR.

**Table 3:** Univariate and multivariate analysis of factors contributing to DSS.

Variables	Univariate		Multivariate	
	Hazard ratio	P value	Hazard ratio	P value
	(95% CI)		(95% CI)	
Age (y.o.)	1.002	0.934		
	(0.961–1.044)			
Gender (Male vs. Female)	1.330	0.503		
	(0.578–3.059)			
Preoperative therapy (yes vs. no)				
Chemotherapy	1.033	0.941		
	(0.436–2.449)			
PTPE	0.853	0.800		
	(0.250–2.917)			
Biliary duct drainage	2.476	0.106		
	(0.826–7.424)			
Preoperative finding of tumor extension to perihilar bile duct*	2.264	0.053		
	(0.99–5.174)			
Preoperative blood examination				
WBC (/μL)	1.000	**0.021**		
	(1.000–1.001)			
Neutrophil (/μL)	1.000	**0.002**		
	(1.000–1.001)			
Lymphocyte (/μL)	0.999	0.080		
	(0.999–1.000)			
Monocyte (/μL)	1.002	0.220		
	(0.999–1.004)			
Platelet (x10^3^/μL)	0.999	0.967		
	(0.973–1.027)			
Total bilirubin (mg/dL)	1.615	0.199		
	(0.778–3.351)			
Total protein (g/dL)	0.788	0.345		
	(0.480–1.293)			
Albumin (g/dL)	0.344	0.067		
	(0.110–1.078)			
Cholesterol (mg/dL)	0.999	0.838		
	(0.988–1.010)			
CRP (mg/dL)	1.208	**0.045**		
	(1.004–1.454)			
CEA (ng/mL)	1.035	**0.044**		
	(1.001–1.069)			
CA19-9 (U/mL)	1.000	0.397		
	(1.000–1.000)			
Preoperative inflammation-based markers				
NLR	1.089	**0.007**		
	(1.024–1.158)			
PLR	1.001	0.192		
	(1.000–1.002)			
LMR	0.737	**0.022**		
	(0.568–0.957)			
**Lymphocyte-to-CRP score: 0 vs. 1 / 2**	**2.629**	**0.022**	**8.566**	**0.008**
	**(1.153–5.997)**		**(1.762–41.636**	
Preoperative inflammation-nutrition-based markers				
PNI	0.919	**0.035**		
	(0.850–0.994)			
**CAR**	**1.727**	**0.048**	**2.762**	**0.017**
	**(1.005–2.969)**		**(1.198–6.367)**	
modified-GPS: 0 vs. 1 / 2	2.983	**0.009**		
	(1.321–6.735)			
CONUT score: 0 / 1 vs. > = 2	1.367	0.446		
	(0.612–3.057)			
Blood loss (g)	1.000	**0.011**		
	(1.000–1.001)			
Operative time (min)	1.003	**0.021**		
	(1.000–1.005)			
Tumor number (single vs. multiple)	1.230	0.661		
	(0.488–3.102			
Maximal tumor diameter (cm)	1.0544	0.533		
	(0.893–1.244)			
TNM (UICC 8th)				
Stage: 0 / 1a / 1b / 2 vs. 3a / 3b / 4	2.419	**0.040**		
	(1.039–5.632)			
**pT: is / 1a / 1b / 2 vs. 3 / 4**	**7.316**	**<0.001**	**16.546**	**0.003**
	**(2.611–20.500)**		**(2.679–102.175)**	
pN: 0 vs. 1	1.513	0.387		
	(0.592–3.865)			
M: 0 vs. 1	0.048	0.733		
	(0.000–1.800618.22)			
Visceral peritoneum invasion (present vs. absent)	7.710	**<0.001**		
	(2.680–2.179)			
**Vascular invasion (present vs. absent)**	**2.274**	**0.047**	**18.459**	**<0.001**
	**(1.011–5.117)**		**(3.990–85.391)**	
Intrahepatic metastasis (present vs. absent)	1.944	0.293		
	(0.563–6.713)			
R0 resection (yes vs. no)	1.237	0.658		
	(0.483–3.166)			
Adjuvant chemotherapy (yes vs. no)	0.549	0.154		
	(0.241–1.253)			

* This finding was evaluated by preoperative computed tomography.

PTPE: percutaneous transhepatic portal vein embolization, WBC: white blood cell, CRP: C-reactive protein, CEA: carcinoembryonic antigen, CA19-9: carbohydrate antigen 19–9, NLR: Neutrophil-to-lymphocyte ratio, PLR: Platelet-to-lymphocyte ratio, LMR: Lymphocyte-to-monocyte ratio, PNI: Prognostic nutritional index, CAR: CRP-to-albumin ratio, GPS: Glasgow prognostic score, CONUT score: Controlling nutritional status score, pT: pathological T, pN: pathological N.

### Comparisons of DSS between resected patients allocated depending on lymphocyte-to-CRP score or CAR and unresected patient

**Fig 2:**
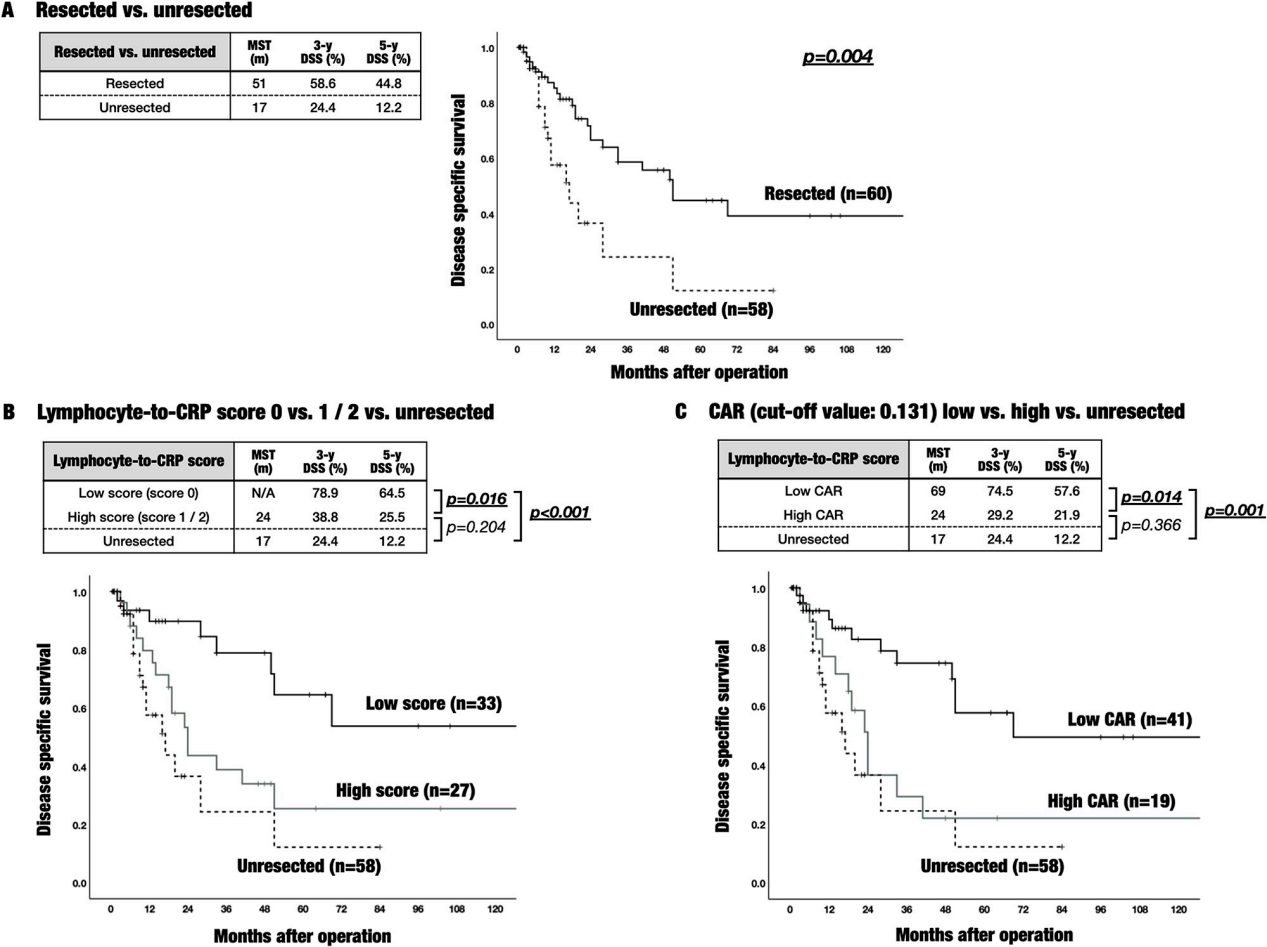
Comparison of DSS between resected patients allocated depending on lymphocyte-to-CRP score or CAR and unresected patient. (A) A comparison of DSS between sixty resected and fifty-eight unresected patients, who were out of surgical indication, for iCCA at our institution between October 2004 and April 2019. Unresected patients had significant poor DSS, compared with resected patients (p = 0.04). In unresected patients, the MST was 17 months, and 3-year / 5-year DSS was 24.4% / 12.2%, whereas in resected patients, the MST was 51 months, and 3-year / 5-year DSS was 58.6% / 44.8%. (B) DSS in patients with a low preoperative lymphocyte-to-CRP-score (score 0) was significantly better than in those with a high score (score 1 or 2) (p = 0.016). In the low score group, the MST did not reach 50% during 120 months, and 3-year / 5-year DSS was 78.9% / 64.5%. In contrast, the MST of DSS in high score group was 24 months, and 3-year / 5-year DSS were 38.8% / 25.5%. DSS in patients with a high lymphocyte-to-CRP score did not show statistical difference, compared with unresected patients (p = 0.204). (C) DSS in patients with a low CAR (< = cutoff value 0.131) was significantly better than in those with a high CAR (p = 0.014). Furthermore, DSS in patients with a high CAR did not show statistical difference, compared with unresected patients (p = 0.366). Abbreviations: DSS: Disease specific survival, iCCA: Intrahepatic cholangiocarcinoma, MST: Median survival time, CRP: C-reactive protein, CAR: CRP-to-albumin ratio.

Fig 2A showed a comparison of DSS between resected and unresected patients for iCCA. Fifty-eight unresected iCCA patients out of surgical indication at our institution had significant poor DSS, compared with resected patients (p = 0.04). In unresected patients, median survival time (MST) of DSS was 17 months, 3-year DSS was 24.4%, and 5-year DSS was 12.2%, whereas in resected patients, MST of DSS was 51 months, 3-year DSS was 58.6%, and 5-year DSS was 44.8%.

Subsequently, based on lymphocyte-to-CRP score and CAR, Kaplan-Meier analysis with a log-rank test was performed in order to compare DSS between good and poor status evaluated by these markers. As shown in Fig 2B, the DSS in patients with a low lymphocyte-to-CRP score (score 0) was significantly better than in those with a high score (score 1 or 2) (p = 0.016). The MST in low score group did not reach 50% during 120 months. In addition to this fine survival rate, 3-year DSS was 78.9%, and 5-year DSS was 64.5%. In contrast, the MST in high score group was 24 months, and 3-year DSS and 5-year DSS were 38.8% and 25.5% respectively. Notably, DSS in patients with a high lymphocyte-to-CRP score did not show statistical difference, compared with the poor DSS in unresected patients (p = 0.204). Regarding CAR, we analyzed the best cut-off values contributing to DSS using the Evaluate Cutpoints, and identified 0.131 as the cut-off values for preoperative CAR. As shown in Fig 2C, DSS in patients with a low CAR (< = 0.131) was significantly better than in those with a high CAR (p = 0.014). The MST in low CAR group was 69 months, and 3-year DSS and 5-year DSS were 74.5% and 57.6% respectively, whereas the MST in high CAR group was 24 months, and 3-year DSS and 5-year DSS were 29.2% and 21.9% respectively. DSS in patients with a high CAR did not show statistical difference, compared with the poor DSS in unresected patients (p = 0.366).

### Patients characteristics and surgical factors in the low and high score groups of lymphocyte-to-CRP score

As shown in Table 4, patients with a low lymphocyte-to-CRP score had significantly lower blood counts of neutrophil and lymphocyte, lower CRP levels, and higher albumin levels than those with a high score (neutrophil; p = 0.023, lymphocyte; p = 0.001, CRP; p < 0.001, albumin; 0.029). Moreover, all inflammatory / immunonutritional markers were significantly better in patients with a low lymphocyte-to-CRP score than in those with a high score (NLR; p < 0.001, PLR; p = 0.004, LMR; p = 0.002, PNI; p = 0.001, CAR; p < 0.001, modified-GPS; p < 0.001, CONUT score; p = 0.007). Amount of blood loss and operative time were significantly lower or shorter in the low score group than in the high score group (blood loss; p = 0.006, operative time; p = 0.024). In terms of histological factors, patients with a low score had significantly smaller maximal tumor diameter and lower rates of pT category 3 to 4 and visceral peritoneum invasion than those with a high score (maximal tumor diameter; p = 0.019, pathological T category; p = 0.041, visceral peritoneum invasion; p = 0.042).

**Table 4:** Patients characteristics and surgical factors in the low and high score groups of lymphocyte-to-CRP score.

Variables	Low score (n = 33)	High score (n = 27)	p value
Age (y.o.)	66 [59–75]	68 [60.5–75]	0.608
Gender (Male / Female)	18 / 15	20 / 7	0.118
Preoperative therapy			
Chemotherapy	7 (25%)	9 (36%)	0.384
PTPE	4 (14%)	2 (8%)	0.499
Biliary duct drainage	1 (4%)	3 (12%)	0.246
Preoperative finding of tumor extension to perihilar bile duct*	12 (36%)	14 (52%)	0.228
Preoperative blood examination			
WBC (/μL)	5210 [4440–6040]	5520 [4570–6940]	0.677
**Neutrophil (/μL)**	**2930 [2440–3660]**	**3610 [2890–5115]**	**0.023**
**Lymphocyte (/μL)**	**1560 [1300–2100]**	**1150 [845–1505]**	**0.001**
Monocyte (/μL)	360 [290–510]	390 [280–510]	0.858
Platelet (x10^3^/μL)	18.1 [14.4–22.9]	18.5 [14.7–22.9]	0.672
Total bilirubin (mg/dL)	0.7 [0.6–0.9]	0.7 [0.5–1.0]	0.626
Total protein (g/dL)	7.1 [6.7–7.4]	7.1 [6.7–7.5]	0.905
**Albumin (g/dL)**	**4.1 [3.9–4.3]**	**3.8 [3.7–4.2]**	**0.029**
Cholesterol (mg/dL)	192 [157–206]	171 [147–207]	0.345
**CRP (mg/dL)**	**0.11 [0.04–0.20]**	**0.77 [0.53–1.42]**	**<0.001**
CEA (ng/mL)	3.9 [2.0–5.2]	3.9 [2.2–8.5]	0.422
CA19-9 (U/mL)	65.1 [26.7–137.0]	79.4 [20.7–331.0]	0.928
Preoperative inflammation-based markers			
**NLR**	**1.82 [1.29–2.34]**	**2.98 [2.34–4.71]**	**<0.001**
**PLR**	**113.4 [76.1–143.6]**	**151.3 [119.7–231.3]**	**0.004**
**LMR**	**4.48 [2.97–5.67]**	**3.04 [2.20–3.86]**	**0.002**
Preoperative inflammation and nutrition-based markers			
**PNI**	**49.6 [46.8–51.7]**	**45.0 [41.0–49.0]**	**0.001**
**CAR**	**0.025 [0.009–0.048]**	**0.191 [0.129–0.381]**	**<0.001**
**modified-GPS: 0 / 1–2**	**33 / 2**	**8 / 19**	**<0.001**
**CONUT score: 0–1 / > = 2**	**21 / 12**	**7 / 20**	**0.004**
**Blood loss (g)**	**620 [320–1327]**	**1670 [1223–3884]**	**0.006**
**Operative time (min)**	**420 [339–500]**	**548 [398–632]**	**0.024**
Tumor number (single / multiple)	24 / 9	23 / 4	0.244
**Maximal tumor diameter (cm)**	**3.8 [2.8–5.0]**	**5.5 [4.0–7.5]**	**0.019**
TNM (UICC 8th)			
Stage: 0–2 / 3–4	24 / 9	14 / 13	0.095
**pT: is–2 / 3–4**	**30 / 3**	**19 / 8**	**0.041**
pN: 0 / 1	26 / 7	19 / 8	0.454
M: 0 / 1	32 / 1	26 / 1	0.885
R0 resection	27 (81%)	20 (74%)	0.469
**Visceral peritoneum invasion**	**3 (10%)**	**8 (32%)**	**0.042**
Vascular invasion	9 (27%)	6 (23%)	0.713
Intrahepatic metastasis	5 (15%)	2 (7%)	0.353
Adjuvant chemotherapy	17 (61%)	11 (44%)	0.224

* This finding was evaluated by preoperative computed tomography.

PTPE: percutaneous transhepatic portal vein embolization, WBC: white blood cell, CRP: C-reactive protein, CEA: carcinoembryonic antigen, CA19-9: carbohydrate antigen 19–9, NLR: Neutrophil-to-lymphocyte ratio, PLR: Platelet-to-lymphocyte ratio, LMR: Lymphocyte-to-monocyte ratio, PNI: Prognostic nutritional index, CAR: CRP-to-albumin ratio, GPS: Glasgow prognostic score, CONUT score: Controlling nutritional status score, pT: pathological T, pN: pathological N.

## Discussion

In the present study, we analyzed the prognostic utility of inflammatory / immunonutritional markers in a multiple comparison among NLR, PLR, LMR, PNI, CAR, modified-GPS, CONUT score, and lymphocyte-to-CRP score, in iCCA patients with surgical resection. Moreover, we firstly revealed that preoperative lymphocyte-to-CRP score was the strongest independent prognostic indicator among inflammatory / immunonutritional markers in resected iCCA patients. Resected iCCA patients with a low lymphocyte-to-CRP score had a significant better prognosis than patients with a high score. Furthermore, there was no statistically difference between resected iCCA patients with a high lymphocyte-to-CRP score and unresected iCCA patients.

Recently, some previous studies reported the prognostic significance of various predictors associated with patient’s status of inflammation, nutrition and immunity for determining oncological outcomes in human malignancies [34] (e.g. hepatocellular carcinoma [35], pancreatic ductal adeno carcinoma [10], and advanced biliary cancer [36]). These inflammatory / immunonutritional markers are categorized into inflammation-based markers such as NLR, PLR, and LMR and inflammation-nutrition-based markers such as PNI, CAR, modified-GPS, and CONUT score. In resected iCCA patients, utilities both of inflammation-based and inflammation-nutrition-based markers had already been confirmed as prognostic indicators. Regarding inflammation-based markers, high levels of NLR [17, 18] and PLR [19], and low levels of LMR [20] contributed to resected iCCA patient’s poor overall survival. As for inflammation-nutrition-based markers, low levels of PNI [21], high levels of CAR [22], and high score of GPS (score 1 / 2) [23] and CONUT score (more than score 2) [24] were associated with poor survival outcome of resected iCCA patients. Among these previous reports, the study by Lin et al. [20] had the most important insight. They compared multiple markers such as modified GPS, NLR, PLR, LMR and PNI in order to identify the strongest predictor in inflammatory / immunonutritional markers; however, this multiple comparison was still not enough, because CAR and CONUT score did not be included. Consequently, the present study analyzed all inflammatory / immunonutritional markers, which had already been confirmed these prognostic significances for resected iCCA patients. Moreover, we added Lymphocyte-to-CRP score, which is a newly reported inflammation-based marker in 2019, to the present multiple comparison.

Lymphocyte-to-CRP score was generated by Okugawa et al. in 2020 [14]. They identified five key factors (neutrophils, lymphocytes, platelets, albumin, and CRP) reflecting systemic inflammation derived from NLR, PLR and CAR, and examined the best combination with highest accuracy to predict oncological outcomes in colorectal cancer patients. As a result, lymphocyte count with CRP were revealed as the strongest predictors in all combinations [15]. Moreover, based on this result, Okugawa et al. [14] developed a new scoring system combining lymphocyte count with CRP, that was named “lymphocyte-to-CRP score”, and exhibited prognostic significance of this score in a large cohort of gastric cancer patients who underwent gastrectomy. Based on multivariate analysis of this study, we identified four predictors, preoperative lymphocyte-to-CRP score, CAR, pT category, and vascular invasion as independent prognostic factors for iCCA patients with surgical resection. In the present study, we focused on preoperative lymphocyte-to-CRP score and CAR among these four factors. This was because we could intervene and improve patient’s status of nutrition and immunity evaluated by inflammatory / immunonutritional factors before operation. Furthermore, to confirm change of these factors provided by our preoperative intervention was very easy. In contrast, regarding pathological factors such as pT category and vascular invasion, it was difficult to intervene and evaluate them certainly prior to operation. Indeed, it is unclear whether neoadjuvant therapy can improve preoperative malignant status in iCCA patients. In cancer immunosurveillance, several studies revealed that peripheral lymphocytes played a crucial role in host cytotoxic-immune response to tumors via identifying and destroying malignant cells [37–39]. Indeed, in patients with poor prognosis, a number of infiltrated lymphocyte around tumor cells was decreased in resected specimens of rectal cancer [16] and lung cancer [40]. High levels of preoperative blood lymphocyte count contributed to better oncological outcome in pancreatic cancer [41] and lung cancer [42]. Regarding serum CRP levels, some previous studies reported that preoperative CRP elevation was significantly associated with a poor prognosis of patients with gastric cancer [43, 44], colorectal cancer [45, 46], and iCCA [47, 48]. CRP is the most representative marker of systemic inflammation and generated by interleukin-6 in a liver [49]. Systemic inflammation produces a lot of proinflammatory cytokines (e.g. interleukin-6, tumor necrosis factor) with consuming a large amount of albumin. Additionally, these cytokines increase vascular permeability, resulting in albumin is leaked transcapillary [50, 51]. Accordingly, systemic inflammation is considered to exacerbate malnutrition via low serum albumin levels in patients with malignancy. Taken together, it was considered that lymphocyte-to-CRP score could reflect host cytotoxic-immune response to tumors, systemic inflammation, and nutritional status in patients with cancer.

In patients with malignancy, several studies exhibited prognostic utilities of various continuous inflammatory / immunonutritional markers including CAR; however, the cut-off value of each marker differed in each study or disease. In contrast, scoring systems such as lymphocyte-to-CRP score should never change these evaluation criteria depending on studies or diseases. Therefore, in a clinical setting, scoring systems are more suitable as biomarkers to provide the same decision, compared with continuous variables. Considering the novel findings from our study, lymphocyte-to-CRP score may represent a feasible scoring system and a great predictor of prognosis in patients with iCCA, especially in a clinical setting.

The present study exhibited that low lymphocyte-to- CRP score group (score 0) had significantly better DSS than high score group (score 1 or 2). In a comparison with characteristics between these two groups, maximal tumor diameter, pT category, and visceral peritoneum invasion were more progressed in patients with a high score. This finding suggested that lymphocyte-to-CRP score might reflect advanced pathological tumor phenotypes. Indeed, Akugul et al. [21] reported a close relationship between low PNI and several advanced tumor phenotypes such as multifocal tumor lesion, poor tumor differentiation, and perineural invasion in resected iCCA patients. In the present study, high lymphocyte-to-CRP score group showed more amount of blood loss and longer operative time than low group. These differences between two groups might be provided from operative difficulty depending on advanced tumor progression.

A noteworthy point of the present study was to firstly demonstrate a comparison between unresected iCCA patients and resected iCCA patients allocated depending on inflammatory / immunonutritional markers. In iCCA, it is clear that resected cases can get better prognosis than unresected cases; therefore, surgical resection is considered the best treatment of improving long-term survival. Indeed, we also confirmed the prognostic significance of surgical resection for iCCA as shown in Fig 2A. Interestingly, there was no significant difference of DSS between resected iCCA patients with a high lymphocyte-to-CRP score and unresected iCCA patients (5-year DSS; 25.5% in the high score resected group vs. 12.2% in the unresected group; p value = 0.204). This result indicated that patients with a high lymphocyte-to-CRP score may not get better prognosis, if they underwent R0 curative resection successfully. Consequently, once an iCCA patient’s status was evaluated poorly by inflammatory / immunonutritional markers, especially lymphocyte-to-CRP score, nutritional therapy should be elected prior to surgical resection in order to improve their status.

In a clinical setting, early intervention with nutritional support is important to improve patient’s status of nutrition and immunity before surgery, especially in elderly patients. Nutritional therapy is thought to attenuate a production of cytokines and normalize host-immune function excessively stimulated through an enhancement of enteric immunity, and is associated with an increase a number of tumor-infiltrating lymphocytes suppressing a cancer progression [52, 53]. In fact, preoperative oral nutritional support using a supplemental liquid diet was reported to reduce both the risk of postoperative infectious complications and the length of hospital stay in pancreatic ductal adenocarcinoma patients undergoing pancreatectomy [54]. As for hepatectomy cases, Yao et al. [55] demonstrated that in patients with hepatocellular carcinoma, preoperative oral nutritional support with enteral nutritional suspension for only 3 consecutive days in addition to normal diet could improve gastrointestinal function, resulting in reducing length of hospital stay. These evidences supported that early intervention with nutritional support may also improve poor status of inflammation, nutrition and immunity in iCCA patients. Additionally, in these patients, the nutritional support should be considered prior to operation.

There were potential limitations in the present study. This study was a retrospective study, and patients were enrolled from a single institution. To overcome this limitations, prospective randomized controlled trials in multicenter are needed. Meanwhile, the present study demonstrated that patients with a high lymphocyte-to-CRP score had more advanced tumor phenotypes. In order to reveal pure effects of preoperative lymphocyte-to-CRP score for a survival outcome, a new trial is needed under matching patient’s tumor phenotypes between high and low score groups.

## Conclusions

In conclusion, preoperative lymphocyte-to-CRP score was the strongest independent prognostic indicator as a result of a multiple comparison among NLR, PLR, LMR, PNI, CAR, modified-GPS, CONUT score, and lymphocyte-to-CRP score in iCCA patients with surgical resection. Furthermore, to our knowledge, the present study was the first report comparing prognosis of resected cases to it of unresected cases in iCCA patients, focusing on a preoperative inflammatory / immunonutritional marker. There was no significant difference between the resected group with a high lymphocyte-to-CRP score and the unresected group.

## Supporting information

S1 Fig Comparison of DSS between resected patients allocated to two groups according to UICC pT or vascular invasion and unresected patients. The present multivariate analysis identified significant prognostic predictors such as pT category (p = 0.003, HR 16.546, 95% CI 2.679–102.175) and pathological vascular invasion (p < 0.001, HR 18.459, 95% CI 3.990–85.391) with preoperative lymphocyte-to-CRP score and CAR for DSS of resected iCCA patients. Based on pT category and venous invasion, we also performed a comparison of DSS using Kaplan-Meier analysis with a log-rank test respectively. (A) We divided resected iCCA patients (n = 60) into pTis to 2 patients and pT3 to 4 patients, depending on whether they had extrahepatic tumor extension or tumor invasion to adjacent organs or not. DSS in patients with pTis to 2 was significantly better than in those with pT3 to 4 (p < 0.001). In patients with pTis to 2, the MST was 69 months, and 3-year / 5-year DSS was 67.8% / 51.8%. In contrast, the MST of DSS in patients with pT3 to 4 was 12 months, and both 3-year and 5-year DSS were 0%. Moreover, DSS in patients with pT3 to 4 did not show statistical difference, compared with unresected patients (n = 58), who were out of surgical indication at our institution in the same period as the resected cases (p = 0.301). (B) DSS in patients without pathological vascular invasion was significantly better than in those with it (p = 0.04). DSS in patients with vascular invasion did not show statistical difference, compared with unresected patients (p = 0.359). Abbreviations: CRP: C-reactive protein, CAR: CRP-to-albumin ratio, DSS: disease specific survival, iCCA: intrahepatic cholangiocarcinoma, pT: pathological T category based on UICC (Union for International Cancer Control) 8th edition, MST: median survival time.
(TIFF)

S1 File (PDF)
