# Supplementary figures and images for: The prognostic impact of lymphocyte-to-C-reactive protein score in patients undergoing surgical resection for intrahepatic cholangiocarcinoma: A comparative study of major representative inflammatory / immunonutritional markers
Source: PLoS One. 2021 Jan 28;16(1):e0245946. doi: 10.1371/journal.pone.0245946 (PMC7842956; doi:10.1371/journal.pone.0245946)

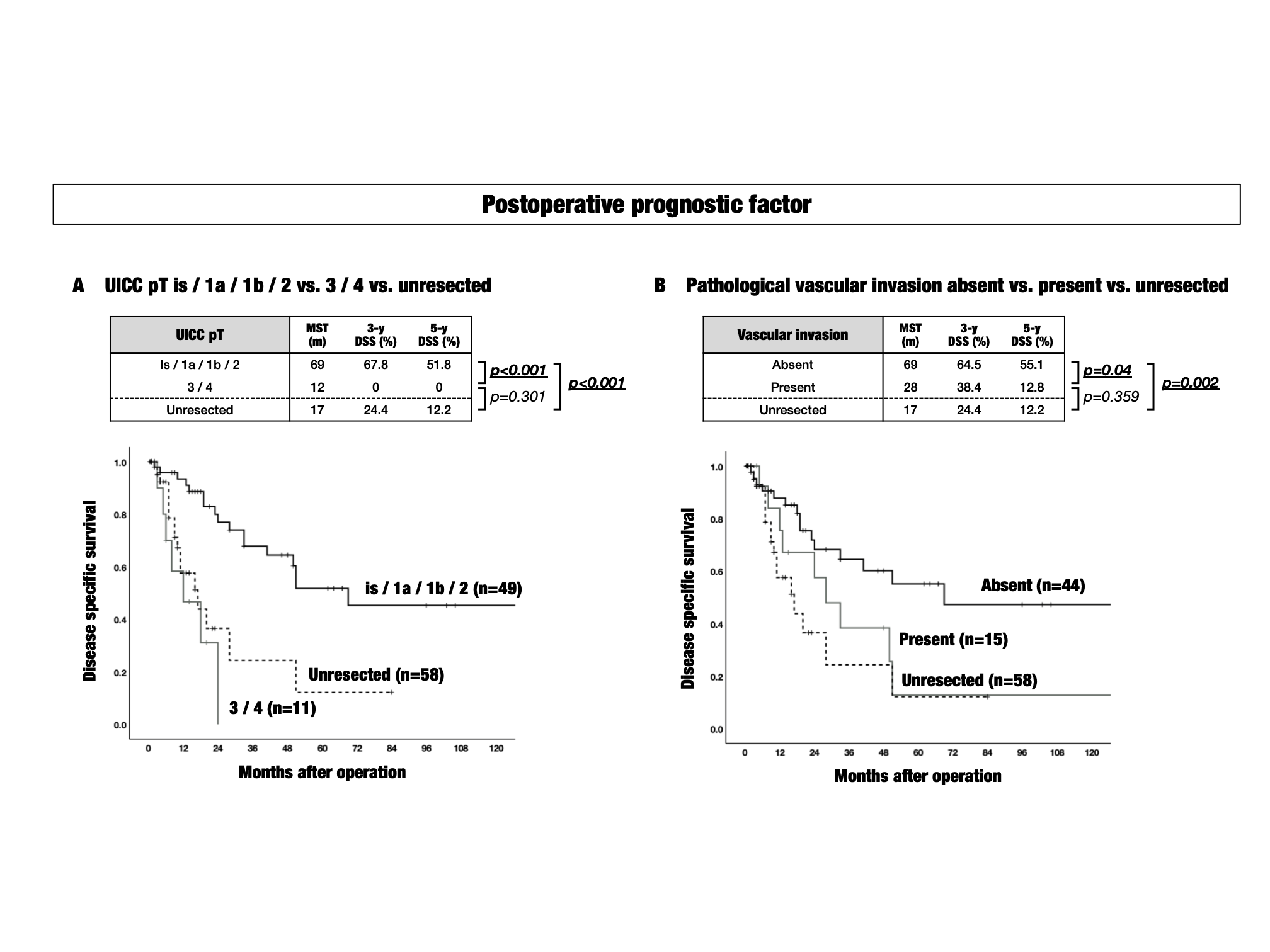

Supplement: S1 Fig — The present multivariate analysis identified significant prognostic predictors such as pT category (p = 0.003, HR 16.546, 95% CI 2.679–102.175) and pathological vascular invasion (p < 0.001, HR 18.459, 95% CI 3.990–85.391) with preoperative lymphocyte-to-CRP score and CAR for DSS of resected iCCA patients. Based on pT category and venous invasion, we also performed a comparison of DSS using Kaplan-Meier analysis with a log-rank test respectively. (A) We divided resected iCCA patients (n = 60) into pTis to 2 patients and pT3 to 4 patients, depending on whether they had extrahepatic tumor extension or tumor invasion to adjacent organs or not. DSS in patients with pTis to 2 was significantly better than in those with pT3 to 4 (p < 0.001). In patients with pTis to 2, the MST was 69 months, and 3-year / 5-year DSS was 67.8% / 51.8%. In contrast, the MST of DSS in patients with pT3 to 4 was 12 months, and both 3-year and 5-year DSS were 0%. Moreover, DSS in patients with pT3 to 4 did not show statistical difference, compared with unresected patients (n = 58), who were out of surgical indication at our institution in the same period as the resected cases (p = 0.301). (B) DSS in patients without pathological vascular invasion was significantly better than in those with it (p = 0.04). DSS in patients with vascular invasion did not show statistical difference, compared with unresected patients (p = 0.359). Abbreviations: CRP: C-reactive protein, CAR: CRP-to-albumin ratio, DSS: disease specific survival, iCCA: intrahepatic cholangiocarcinoma, pT: pathological T category based on UICC (Union for International Cancer Control) 8th edition, MST: median survival time. (TIFF) [file pone.0245946.s001.tiff]
